# HIV stigma: perceptions from HIV-positive and HIV-negative patients in a community dental clinic

**DOI:** 10.15171/joddd.2016.042

**Published:** 2016-12-21

**Authors:** Steven Toth, Jill A. York, Nicholas DePinto

**Affiliations:** ^1^Department of Community Health, Rutgers School of Dental Medicine, Rutgers , The State University of New Jersey, New Jersey, USA

**Keywords:** Attitudes, HIV/AIDS, mental health, public health, self-perception, stigma

## Abstract

***Background. ***In the medical sense, stigma has been defined as the collection of negative attitudes and beliefs that are directed at people living with a particular condition or disease process. A cohort study was conducted to explore the HIV stigma that is perceived by HIV-positive individuals versus that perceived by the general population within a community-based dental clinic.

***Methods.*** Two separate and independent cross-sectional surveys, the Berger Stigma Scale and the Rutgers-Modified Berger Stigma Scale, were employed in order to analyze the stigma factors of an HIV-positive population versus an HIV-negative general population, respectively. The HIV stigma factors studied included personalized stigma, disclosure concerns, negative self-image, and concern with public attitudes.

***Results.*** The total stigma scale scores for the studied HIV-positive population were significantly lower than the total stigma scale scores for the studied HIV-negative population (P < 0.05).

***Conclusion.*** Interestingly, there is a misplaced expectation by the general population that HIV-positive individuals experience more stigma than the HIV-positive population in the clinic actually reported. Interventions to reduce HIV stigma should be an integral component of comprehensive care for all patients.

## Introduction

Stigma in a medical setting can be succinctly defined as the combined group of all negative beliefs and preconceptions that are aimed at people living with a particular condition or disease process.^1,2^ This stigma that is projected upon the affected population represents a destructive social marker which adversely harms that group’s self-image.^3^ The negative umbrella of stigma is often compared to racism and discrimination, and the shadow stigma casts is known for the persistent struggle it causes in the affected.^2,4^ Living with a stigmatizing condition can significantly degrade the quality of life of an individual, potentially leading to social withdrawal, diminished self-worth, and depression.^5^ Unfortunately, as a result of stigma, an affected individual’s condition or disease process may eventually come to wholly consume and identify that person.^3,6^ Regardless of the fact that public knowledge and awareness of HIV/AIDS has markedly improved since the epidemic began decades ago, it is generally agreed that a definite social stigma is still associated with the disease.^7^The general population in the United States believes that stigma is still an issue for people living with HIV/AIDS, and the authors determined to test whether or not the HIV positive population feels the same way as the general public expects them to feel.^8,9^

Methods have been developed to measure the stigma that is perceived by those infected with HIV, with the Berger Stigma Scale (BSS)being considered as one of the most effective and reliable.^10,11^ Separately, public reactions toward people infected with HIV have also been examined, leading to the understanding that a sizable portion of the general population still holds negative beliefs and preconceptions toward those with HIV/AIDS.^12,13^ Widespread studies have been conducted regarding the stigma perceived by HIV-positive individuals, as well as the stigma that the general population feels toward those with HIV/AIDS; nonetheless, limited studies have been carried out on the comparison between these two populations within the same geographic and demographic settings.^14,15^ Recognizing this disconnect, we sought to fill this gap by measuring the stigma perceived by people with HIV compared to the stigma projected by the general population. This was examined by employing the BSS for HIV-positive individuals and the Rutgers-Modified Berger Stigma Scale (RMBSS) for the HIV-negative population. Since the BSS was designed specifically for persons infected with HIV, the RMBSS was created as its congruent analog, maintaining every question, modified only in language so as to reflect a non-infected individual’s opinion. Both tools measured stigma in four factors: personalized stigma, disclosure concerns, negative self-image, and concern with public attitudes and also assigned a total stigma score.^10^

## Methods

All procedures performed in studies involving human participants were in accordance with the ethical standards of the institutional and/or national research committee and with the 1975 Helsinki Declaration and its later amendments. The protocol was approved by the UMDNJ-Stratford Campus/Rutgers Institutional Review Board.

### Question development 

The BSS was designed at the College of Nursing, University of Illinois in Chicago in 2001.The BSS is validated, standardized, and widely used to measure stigma in the HIV-positive population. It measures stigma perceived by people living with HIV, and was developed based on the literature on stigma and psychosocial aspects of having HIV.^10^ The 40 items of the BSS focus on experiences, feelings, and opinions as to how people living with HIV feel and how they are treated (Table 1). The person living with HIV responds to these items using a four-point Likert-type scale (strongly disagree, disagree, agree, strongly agree).Higher values indicate a greater level of agreement with each item. Rutgers School of Dental Medicine (RSDM) developed a derivative, Rutgers Modified Stigma Scale (RMBSS) in 2010, which was designed to measure stigma projected by people who do not have AIDS. The 40 items of the RMBSS focus on experience, feelings, and opinions as to how the general population perceive and treat those who are HIV-positive (Table 2). The person not living with HIV responds to these items using a four-point Likert-type scale (strongly disagree, disagree, agree, or strongly agree). Higher values indicate a greater level of agreement with each item. Test-retest correlation for the survey instruments were analyzed, measuring 0.92 for the BSS survey and 0.82 for the RMBSS survey. Both tools have the same score ranges. The total HIV score can range from 40 to 160, the personalized stigma score can range from 18 to 72, the disclosure concern score can range from 10 to 40, the negative self-image score can range from 13 to 52, and the public attitude score can range from 20 to 80; the higher the score, the higher and more severe the stigma.

**Table 1:** Berger Stigma Scale questions

1. In many areas of my life, no one knows I have HIV.
2. I feel guilty because I have HIV.
3. People's attitudes about HIV make me feel worse about myself.
4. Telling someone I have HIV is risky.
5. People with HIV lose their jobs when their employers find out.
6. I work hard to keep my HIV a secret.
7. I feel I'm not as good a person as others because I have HIV.
8. I never feel ashamed of having HIV.
9. People with HIV are treated like outcasts.
10. Most people believe a person who has HIV is dirty.
11. It is easier to avoid new friendships than worry about telling someone that I have HIV.
12. Having HIV makes me feel unclean.
13. Since learning I have HIV, I feel set apart and isolated from the rest of the world.
14. Most people think a person with HIV is disgusting.
15. Having HIV makes me feel I'm a bad person.
16. Most with HIV are rejected when others find out.
17. I am very careful who I tell that I have HIV.
18. Some people who know I have HIV have grown more distant.
19. Since learning I have HIV, I worry about people discriminating against me.
20. Most people are uncomfortable around someone with HIV.
21. I never feel I need to hide the fact I have HIV
22. I worry that people may judge me when they learn I have HIV.
23. Having HIV in my body is disgusting to me.
24. I have been hurt by how people reacted to learning I have HIV.
25. I worry that people who know I have HIV will tell others.
26. I regret having told some people that I have HIV.
27. As a rule, telling others that I have HIV has been a mistake.
28. Some people avoid touching me once they know I have HIV.
29. People I care about stopped calling after learning I have HIV.
30. People have told me that getting HIV is what I deserve for how I lived my life.
31. People close to me are afraid others will reject them if it becomes known that I have HIV.
32. People don't want me around their children once they know I have HIV.
33. People have physically backed away from me when they learn I have HIV.
34. Some people act as though it's my fault I have HIV.
35. I have stopped socializing with people because of their reactions to my having HIV.
36. I have lost friends by telling them I have HIV.
37. I have told people close to me to keep the fact that I have HIV a secret.
38. People who know I have HIV tend to ignore my good points.
39. People seem afraid of me once they learn I have HIV.
40. When people learn you have HIV, they look for flaws in your character.

The Berger HIV Stigma Scale utilizes forty questions to quantify stigma that HIV infected individuals perceive. It was originally published by Berger in “Measuring stigma in people with HIV: psychometric assessment of the HIV stigma scale.”

**Table 2:** Rutgers Modified Berger Stigma Scale (RMBSS)questions

1. In many areas of people with HIV's lives, no one knows they have it.
2. People feel guilty because they have HIV.
3. People's attitudes about HIV makes those with HIV feel worse about themselves.
4. Telling someone that they have HIV is risky.
5. People with HIV lose their jobs when their employers find out.
6. People with HIV work hard to keep it a secret.
7. People with HIV feel they are not as good a person as others because they have HIV.
8. People with HIV never feel ashamed of having HIV.
9. People with HIV are treated like outcasts.
10. Most people believe a person who has HIV is dirty.
11. It is easier to avoid new friendships than worry about telling someone that they have HIV.
12. Having HIV makes someone feel unclean.
13. After learning they have HIV, people feel set apart and isolated from the rest of the world.
14. Most people think a person with HIV is disgusting.
15. Having HIV makes someone feel they are a bad person.
16. Most with HIV are rejected when others find out.
17. People with HIV are very careful who they tell.
18. People grow more distant when they know someone has HIV.
19. After learning they have HIV, people worry about others discriminating against them.
20. Most people are uncomfortable around someone with HIV.
21. People with HIV never feel the need to hide the fact they have HIV
22. People with HIV worry that they may be judged when others learn they have HIV
23. Having HIV in one's body is disgusting
24. People with HIV have been hurt by how others reacted to learning they have HIV
25. People with worry that someone who knows will tell others.
26. People with HIV regret telling some that they have HIV.
27. As a rule, telling others that they have HIV is a mistake.
28. Some people avoid touching others once they know they have HIV.
29. People that care about someone with HIV stop calling after learning that person has HIV.
30. People say that getting HIV is what someone deserves for how they lived their life.
31. Some people are afraid that others will reject them if it becomes known that someone close to them has HIV.
32. People don't want someone with HIV around their children.
33. People have physically backed away from someone when they learn they have HIV.
34. Some people act as though it’s their own fault they have HIV.
35. People with HIV have stopped socializing with some people because of their reactions to their having HIV.
36. People have lost friends by telling them they have HIV.
37. People with HIV tell those close to them to keep the fact that they have HIV a secret.
38. People who know someone who has HIV tends to ignore their good points.
39. People seem afraid of someone once they learn they have HIV.
40. When people learn you have HIV, they look for flaws in their character.

The RMBSS utilizes 40 questions to quantify the stigma that HIV negative individuals perceive about individuals that are HIV positive. It is an adapted version of the Berger Stigma Scale.

### Recruitment and consent

During the course of routine dental visits, active patients of the Rutgers extramural dental clinics in Galloway and Somerdale, New Jersey, were invited to participate. At the time of data collection, 18% of the active patient pool of these clinics were HIV-positive. Within this patient pool, two populations were identified. The first consisted of patients aged 25 through 64, who were HIV-positive, and the second consisted of patients from the general population, aged 25 through 64, who were HIV-negative. All the participants gave informed consent in this institutional review board-approved project. Any patient under the age of 25 or over the age of 65 was ineligible to participate. Additionally, any patient aged 25 through 65 that could not read English was also excluded.

### Procedure

A cross-sectional study was conducted in the two RSDM extramural clinics. Both of these clinics have been providing oral health services for individuals infected with HIV and have received Ryan White Funding for over 25 years. Intake receptionists distributed the appropriate surveys to patients based on their medical history as they checked in for their appointments. Data was collected for both populations via the surveys. Surveys were completed voluntarily and anonymously, placed in sealed envelopes, and dropped into a secured receptacle in the dental clinic waiting room.

### Analysis

A total enrollment of 200 participants (100 HIV-positive patients and 100 HIV-negative patients) provided a 95±9% CI based upon the number of active unduplicated AIDS patients enrolled in the extramural clinics.

Mean scores, standard deviations, medians, variances, and averages of absolute deviations were calculated for each of the four stigma factors (personalized stigma, disclosure concerns, negative self-image, and concern with public attitudes) and the total HIV stigma score for both AIDS and non-AIDS populations. The primary analysis compared results from the BSS to the RMBSS.

## Results

### Participant characteristics

Population analysis of the two enrolled cohorts reflected the demographic profile of the local populations of both groups, and illustrated significant differences between the HIV-positive and HIV-negative groups.^16,17^ Of the 100 enrolled HIV-infected participants, those with self-reported race/ethnicity as Black/African American constituted nearly half of the received surveys (45%) (Table 3). Three quarters of this cohort’s respondents were male (72%) and the remainder were female (27%), with one transgender respondent.

**Table 3:** Demographic profile of participants

**Age**	**Number of HIV+ Respondents**	**Number of HIV- Respondents**
**25‒34**	16	23
**35‒44**	21	19
**45‒55**	42	34
**56‒64**	21	24
**Gender**		
**Male**	72	49
**Female**	27	50
**Transgender**	1	1
**Ethnicity**		
**White**	34	81
**African American**	45	11
**Hispanic**	16	1
**Asian**	5	7

The demographic profiles of survey participants reflect the demographic profiles of the local community. Minorities are represented more heavily in the HIV infected cohort.

A clear majority of the 100 enrolled non-HIV-infected cohort self-reported race/ethnicity as Caucasian (81%) (Table 3).The non-AIDS cohort was evenly split between male and female respondents (49% and 50%, respectively), with one transgender participant.

Two thirds of both groups reported to be between the ages of 44 and 64 years.

### Stigma scale results

The stigma scale scores of the two cohorts, with the Berger Stigma Scale used for the HIV-positive population and the Rutgers-Modified Stigma Scale used for the HIV-negative population were tabulated and analyzed (Tables 4 and 5).The difference between the average Total HIV Stigma Score for the HIV-negative cohort using the RMBSS and HIV-positive cohort using the BSS was statistically significant (P < 0.05), scoring 110.5 versus 97.2, respectively. Additionally, all individual stigma factors were higher in the HIV-negative cohort, including personalized stigma, disclosure concerns, negative self-image, as well as public attitudes. The disclosure concern subscale represented the most significant deviation between the two groups scoring 60.1 versus 28.0 for the HIV-negative and HIV-positive cohorts, respectively.

**Table 4:** Berger Stigma Scale scores for the studied HIV-positive population

**Analysis**	**Personalized Stigma**	**Disclosure Concerns**	**Negative Self-image**	**Concern with Public Attitudes about People with HIV**	**Total HIV Stigma Score**
**Mean Scores**	41.0	28.0	29.4	48.5	97.2
**Standard Deviation**	12.1	5.9	813	12.4	23.2
**Median**	40.0	28.0	29.0	48.0	95.0
**Variance**	145.8	34.7	66.1	154.9	535.7
**Avg of Abs. Dev.**	8.9	4.7	6.0	9.6	17.4

The four stigma subscales and total stigma scores of the HIV infected population as recorded from the Berger Stigma Scale.

**Table 5:** Rutgers Modified Berger Stigma Scale scores for the studied HIV-negative population

	**Personalized Stigma**	**Disclosure Concerns**	**Negative Self-image**	**Concern with Public Attitudes about People with HIV**	**Total HIV Stigma Score**
**Mean Scores**	49.3	60.1	34.9	53.9	110.5
**Standard Deviation**	7.4	4.1	5.0	8.5	15.6
**Median**	49.0	29.0	34.0	54.0	108.0
**Variance**	54.8	16.9	25.0	72.3	246.9
**Avg of Abs. Dev.**	5.6	3.1	3.9	6.6	12.1

The four stigma subscales and total stigma scores of the general population as recorded from the Rutgers Modified Berger Stigma Scale and their associated analysis.

## Discussion

There is an exaggerated expectation in the general population that HIV-positive individuals experience a certain degree of stigma. To remedy this, there must be interventions to prevent HIV-related stigma.^18^ Community-based organizations can take steps to improve the public’s lack of understanding of HIV and increase acceptance of HIV-positive individuals. Through education, we have a responsibility to decrease the public’s rejection of people with HIV (personalized stigma), foster a community of unconditional regard for an individual’s status (disclosure concerns), eliminate any projection of inferiority towards HIV-positive individuals (negative self-image), and change the public’s preconceptions about people with HIV (concern with public attitudes).

This study entirely relied on self-reported survey—a type of instrument known to have inherent limitations with regards to accuracy and reliability.^19^The results are dependent upon the honesty of respondents, their objective introspective ability, as well as their response bias.^19^ To minimize this limitation, surveys were completed anonymously and privately. It might be surmised that the HIV-positive participants that took part in this study did not experience a significant degree of stigma. However, it can be noted that this cohort might have been biased towards a sense of approval and acceptance—the community-based dental clinic that disseminated this study takes steps to minimize stigma perceptions among its patients. Support group presentations, literature distribution, poster presentations, and open dialogue and discussion about HIV are pillars of the clinics. The lower stigma perception of participants in this dental clinic might bias results.

Although the samples differed in their demographic makeup, the populations studied were representative of the New Jersey demographic profiles for each group surveyed: New Jersey’s HIV-positive and HIV-negative populations. Further studies and analyses could be considered to rule out possible demographic skewing of our results to minimize the impact that New Jersey’s HIV-positive population is more demographically diverse than its general population.

## Conclusion

This study demonstrates that decades into the HIV epidemic, there is still a misplaced stigma projected towards individuals with HIV by the general population. Our findings reaffirm the need to educate the public about minimizing projected stigma in all its forms: personalized stigma, disclosure concerns, negative self-image, and concern with public perceptions. To that end, community dental clinics must open a dialogue about HIV with *all* patients, not just those infected with HIV. Doing so will bring HIV out in the public consciousness and reduce the negative beliefs and preconceptions aimed at people living with HIV.

## Acknowledgements

Technical assistance was provided by Brittni Smith.

## Authors’ information

STEVEN TOTH, DMD is an Assistant Professor in the Department of Community Health at the Rutgers School of Dental Medicine and the Clinical Director of the Ryan White Program at the Rutgers School of Dental Medicine, University Dental Center at Galloway. JILL YORK, DDS, MAS is an associate professor in the Department of Community Health and the Assistant Dean of Extramural Clinics at the Rutgers School of Dental Medicine. NICHOLAS DEPINTO, DMD is an assistant professor in the Department of Community Health at the Rutgers School of Dental Medicine and the Dental Director of the Rutgers School of Dental Medicine, University Dental Center at Galloway.

## Authors’ contributions

ST was responsible for the study conception and design, data collection and analysis, and drafting and revision of the manuscript. JY was responsible for the study conception and design, data collection and analysis, and drafting and revision of the manuscript. ND was responsible for the study conception and design, data collection and analysis, and drafting and revision of the manuscript. All of the authors have read and approved the final manuscript.

## Funding

This project was supported by the Health Resources and Services Administration (HRSA) of the U.S. Department of Health and Human Services (HHS) under the Community Based Dental Partnership Program grant number H65HA26354 with funding of $364,172 and no funding from nongovernmental sources. This information or content and conclusions are those of the authors and should not be construed as the official position or policy of, nor should any endorsements be inferred by HRSA, HHS or the U.S. Government. Funding played no role in the design of the study, collection, analysis, or interpretation of data.

## Competing interests

The authors declare no competing interests with regards to the authorship and/or publication of this article.

## Ethical approval

All procedures performed in studies involving human participants were in accordance with the ethical standards of the institutional and/or national research committee and with the 1975 Helsinki Declaration and its later amendments. The protocol was approved by the UMDNJ-Stratford Campus/Rutgers Institutional Review Board.

## References

[1] Brimlow D, Cook J, Seaton R. Stigma and HIV/AIDS: A review of the literature. US Department of Health and Human Services Health Resources and Services Administration, HIV/AIDS Bureau, 2003.

[2] Hatzenbuehler M, Phelan J, Link B (2013). Stigma as a fundamental cause of population. health inequalities.

[3] Goffman E (1963). Stigma: notes on the management of spoiled identity.

[4] O’Brien O, Khan S (1996). Stigma and racism as they affect minority ethnic communities. Crossing borders: Migration, ethnicity and AIDS.

[5] Varni S, Miller C, McCuin T (2012). Disengagement and engagement coping with HIV/AIDS stigma and psychological well-being of people with HIV/AIDS. J Soc Clin Psychol.

[6] Richeson J, Shelton N. Intergroup dyadic interactions. In: Dovidio J, Hewstone M, Glick P, editors.The SAGE handbook of prejudice, stereotyping and discrimination.London, Sage Publications, 2010.

[7] Kaiser Family Foundation, 2011. HIV/AIDS at 30: A public opinion perspective. (KFF Pub. No. [KFF] 8186.)Menlo Park, CA: Kaiser Family Foundation, 2011.

[8] Klein E. United States public opinion toward HIV/AIDS: Perceptions of risk, bias, and government spending. New York, NY: Gay Men’s Health Crisis, 2009.

[9] Rohn E, Sankar A, Hoelscher C, Luborsky M, Parise MH (2006). How do social-psychological concerns impede the delivery of care to people with HIV? Issues for dental education. J Dent Educ.

[10] Berger B, Ferrans C, Lashley F (2001). Measuring stigma in people with HIV: psychometric assessment of the HIV stigma scale. Res Nurs Health.

[11] Wright K, Naar-King S, Lam P, Templin T, Frey M (2007). Stigma scale revised: reliability and validity of a brief measure of stigma for HIV + youth. J Adolesc Health.

[12] Herek GM, Capitanio JP (1993). Public Reactions to AIDS in the United States: A Second Decade of Stigma. Am J Pub Health.

[13] Emlet C (2005). Measuring stigma in older and younger adults with HIV/AIDS: an analysis of an HIV stigma scale and initial exploration of subscales. Res Soc Work Pract.

[14] Kerr JC, Valois RF, Diclemente RJ, Fletcher F, Carey MP, Romer D (2014). HIV-related stigma among African-American youth in the northeast and southeast US. AIDS Behav.

[15] Rutledge SE, Whyte J, Abell M, Brown KM, Cesnales NI (2011). Measuring stigma among health care and social service providers: The HIV/AIDS Provider Stigma Inventory. AIDS Patient Care STDS.

[16] Public Health Services Branch Division of HIV, STD and TB Services.HIV/AIDS Report. Trenton, NJ:New Jersey Department of Health, 2013.

[17] U.S. Census Bureau: State and County QuickFacts. Trenton, NJ: U.S. Census Bureau, 2014.

[18] Sohini S, Banks B, Jonas D, Shandor M, Smith GC (2011). HIV Interventions to Reduce HIV/AIDS Stigma. A SystematicReview, AIDS Behav.

[19] Austin EJ, Deary IJ, Gibson BJ (1998). McGregor MJ, DentJB Individual response spread in self-report scales: personality correlations and consequences. Pers Individ Dif.

